# Arteriolar and Venular Remodeling Are Differentially Regulated by Bone Marrow-Derived Cell-Specific CX3CR1 and CCR2 Expression

**DOI:** 10.1371/journal.pone.0046312

**Published:** 2012-09-24

**Authors:** Joshua K. Meisner, Ji Song, Richard J. Price

**Affiliations:** 1 Department of Biomedical Engineering, University of Virginia, Charlottesville, Virginia, United States of America; 2 Department of Radiology, University of Virginia, Charlottesville, Virginia, United States of America; 3 Department of Radiation Oncology, University of Virginia, Charlottesville, Virginia, United States of America; University of Illinois at Chicago, United States of America

## Abstract

The chemokine receptors CCR2 and CX3CR1 are critical for the recruitment of “inflammatory” and “resident” monocytes, respectively, subpopulations that differentially affect vascular remodeling in atherosclerosis. Here, we tested the hypothesis that bone marrow-derived cell (BMC)-specific CCR2 and CX3CR1 differentially control venular and arteriolar remodeling. Venular and arteriolar lumenal remodeling were observed by intravital microscopy in mice with either CCR2 or CX3CR1 deficient BMCs after implantation of a dorsal skinfold window chamber, a model in which arterioles and venules lumenally enlarge in wild-type (WT) mice. Arteriolar remodeling was abolished in mice with either CCR2 or CX3CR1-deficient BMCs. In contrast, the loss of CX3CR1 from BMCs, but not CCR2, significantly reduced small venule remodeling compared to WT controls. We conclude that microvascular remodeling is differentially regulated by BMC-expressed chemokine receptors. Both CCR2 and CX3CR1 regulate arteriole growth; however, only BMC-expressed CX3CR1 impacts small venule growth. These findings may provide a basis for additional investigations aimed at determining how patterns of monocyte subpopulation recruitment spatially influence microvascular remodeling.

## Introduction

Bone marrow derived cells (BMCs), and monocytes in particular, regulate vascular remodeling. However, multiple functionally distinct monocyte subpopulations exist [1]. The two best characterized subpopulations can be defined by their relative expression of chemokine receptors CCR2 and CX3CR1 in mice and surface markers CD14 and CD16 in humans [2], [3]. The “inflammatory” CCR2^hi^CX3CR1^lo^ (CD14^+^CD16^−^) subset is mobilized from bone marrow and splenic reservoirs through systemic signaling and recruited to sites of active inflammation through local expression of chemokine CCL2 (MCP-1) [4]–[6]. In contrast, cells in the “resident” CCR2^lo^CX3CR1^hi^ subset (CD14^lo^CD16^+^) have a longer intravascular and tissue presence and exhibit a venous patrolling behavior [1], [7].

Recently, it has been shown that both CCR2- [8] and CX3CR1-dependent [9] BMC recruitment influence microvascular remodeling induced by skin-wound healing. Interestingly, in the context of another vascular remodeling scenario (i.e. atherosclerosis), “inflammatory” and “resident” monocytes exhibit different functional roles in remodeling [10], [11]. Given this background, as well as the recent emphasis on both CCR2-mediated recruitment through arterial signaling during arteriogenesis [12], [13] and CX3CR1-mediated recruitment through venous patrolling [7], we hypothesize that monocytes that are recruited to tissue via CCR2 and CX3CR1 play differential roles in arteriolar and venular microvascular remodeling [14]. Here, we tested this hypothesis by examining the remodeling of arterioles and venules through time in dorsal skinfold window chambers (DSFWCs) implanted on chimeric mice that were generated by transplanting bone marrow from either CCR2^−/−^ or CX3CR1^−/−^ donors into wild type (WT) hosts.

## Results

### Time-course of Recruitment of CCR2- and CX3CR1-Positive Leukocyte Subpopulations in the Dorsal Skinfold Window Chamber (DSFWC)

**Figure 1:**
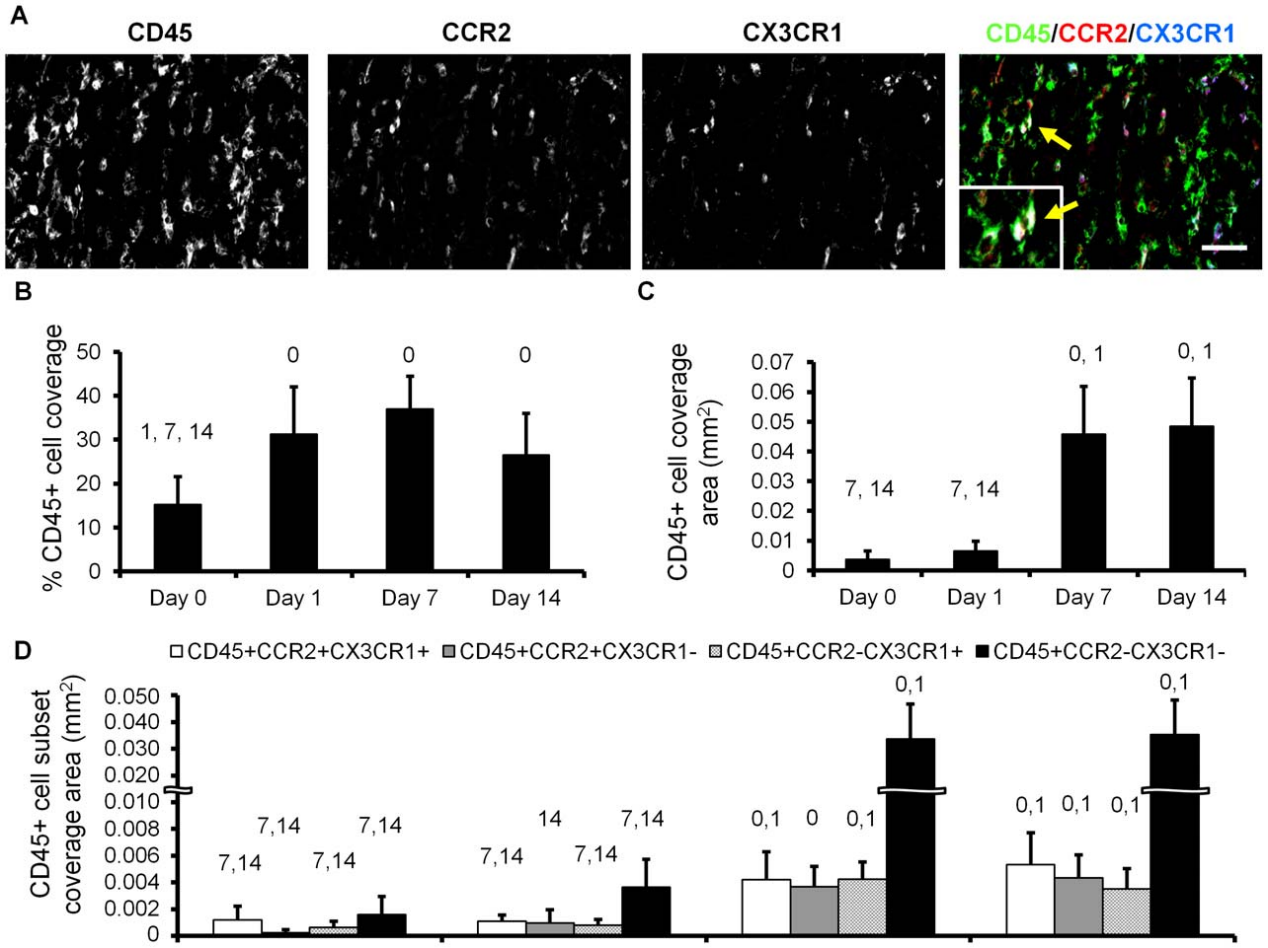
Immunolabeling for CD45, CCR2, and CX3CR1 in dorsal skinfold window chambers. A) Representative images from day 7 window chamber, arrows indicate triple-marker colocalization in the merged panel with zoomed inset. Scale bar is 50 µm. B,C) Quantification of CD45+ leukocyte coverage as absolute (C) and percentage (B) of cross sectional tissue area. D) Analysis of colocalization of CD45, CCR2, and CX3CR1 from day 0 to day 14 [^0, 1, 7, 14^ indicates p<0.05 versus day 0, 1, 7, 14, respectively within cell type coverage, n>8 sections from 2–3 mice per time point].

DSFWCs were implanted on WT mice as previously described [8]. CD45+ leukocyte infiltration (Figure 1A, left column) was assessed in WT DSFWC tissue at 0, 1, 7, and 14 days post-implantation (non-specific staining was not present in isotype controls, Figure S1). Within the first 24 hours, CD45+ cell density increased by 105% and remained significantly elevated through Day 14 (Fig. 1B). Absolute CD45+ cell area increased substantially from Day 1 to Day 7 (Fig. 1C); however, this did not translate into a change in percent CD45+ cell coverage (Fig. 1B) because tissue thickness increases significantly from Day 1 to Day 7 [8].

The colocalization of CCR2 and CX3CR1 with CD45 [Fig. 1A (right panel) & Figure 1D] was assessed to track all bone-marrow derived leukocyte (CD45+) subpopulations in DSFWC tissue through time in wild-type mice, where both CCR2 and CX3CR1 are expressed. We observed a significant accumulation of all CCR2^+/−^/CX3CR1^+/−^/CD45^+^cellular populations from day 0 to day 14, with these changes primarily occurring between Days 1 and 7 (Figure 1D). No significant differences occurred within the first 24 hours (Fig. 1D). Further, the recruited leukocyte populations were stable during the observed vascular remodeling period, with no significant changes in accumulation from day 7 to day 14 (Figure 1D). Mac3^+^ immunofluorescence indicated that the recruited CCR2^+^ and CX3CR1^+^ leukocyte populations were predominantly of macrophage origin (Figure S2).

### BMC-Expressed CX3CR1 and CCR2 Differentially Regulate Arteriolar and Venular Remodeling in the DSFWC

**Figure 2:**
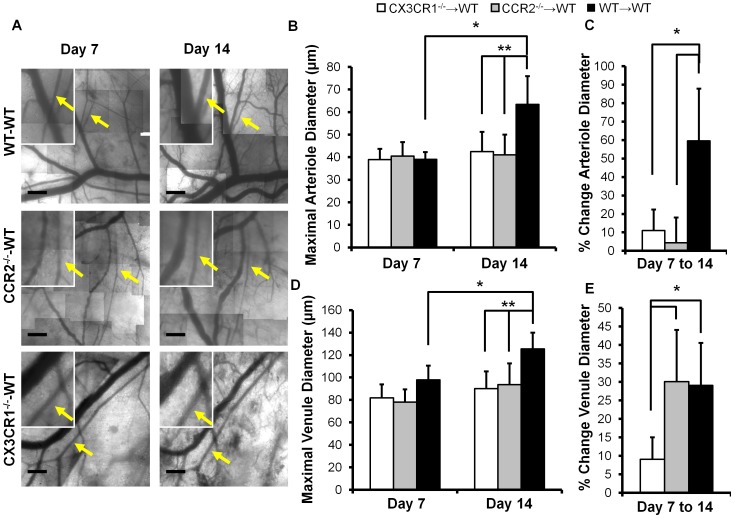
Arteriolar and venule remodeling in dorsal skinfold window chamber is differentially altered by bone marrow specific loss of CCR2 and CX3CR1. A) Representative images of window chamber regions at day 7 and 14 from experimental groups. Insets are higher magnification images of selected regions (arrows indicate arterioles, scale bar is 300 µm). Mean arteriole (B) and venule (D) diameters were quantified in all microvascular segments for each mouse in experimental groups at day 7 and 14 (*, indicates significant difference between days within a group, and ** between groups within a day, p<0.05). Average percent change in diameter from day 7 to 14 was quantified in all arteriole (C) or venule (E) segments per mouse and averaged across experimental groups (*, indicates p<0.05 significant difference between groups). [Data represents n = 7 WT→WT, n = 4 CX3CR1^−/−^→WT, and n = 6 CCR2^−/−^→WT mice per group].

**Figure 3:**
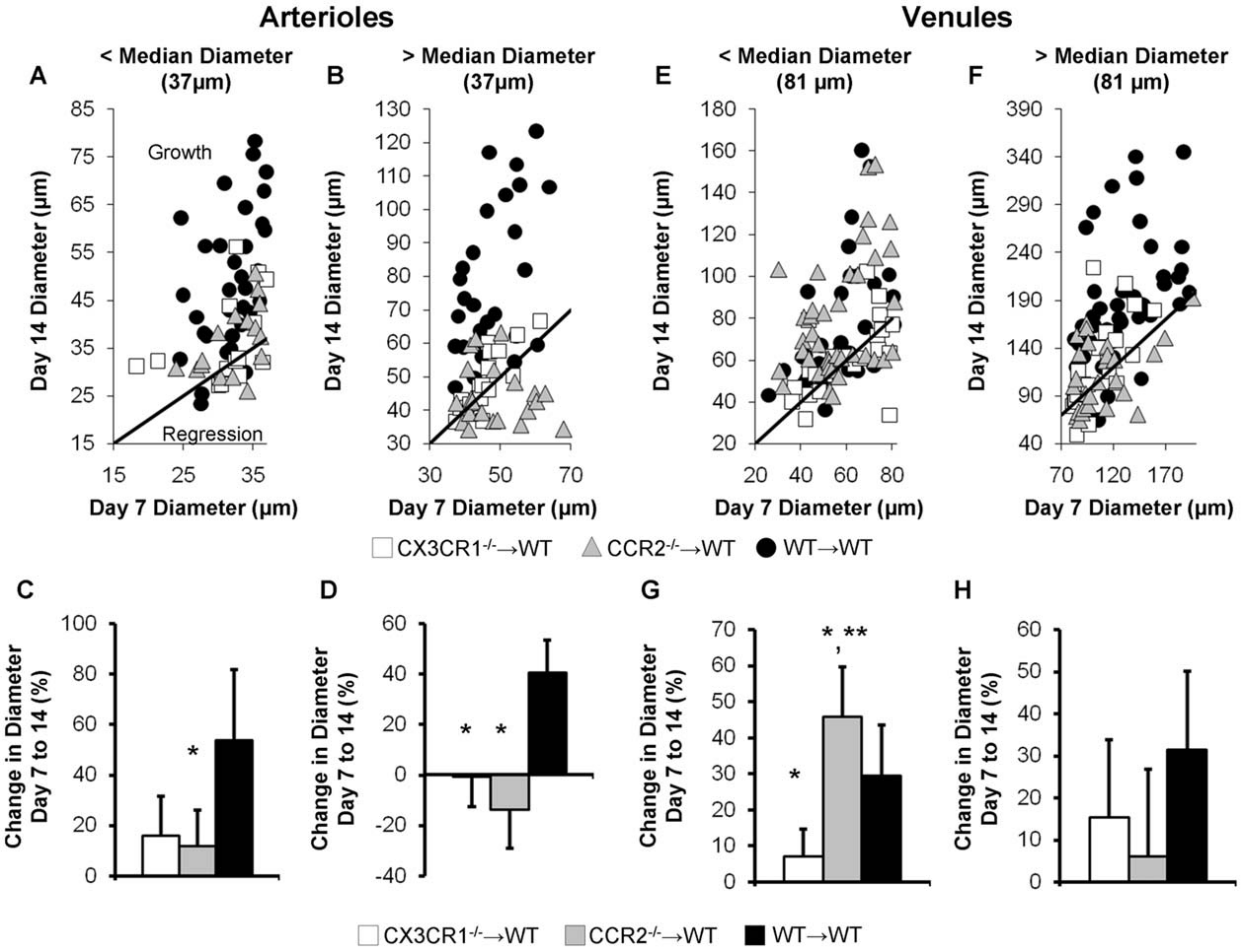
Influence of initial vessel diameter on remodeling. Arteriole (A–D) and venule (E–H) remodeling data were split into initial day 7 diameters above and below median diameter. Illustration of growth and regression for CX3CR1^−/−^→WT, CCR2^−/−^→WT, and WT→WT mice (A, B, E, F). Quantification of percent change in maximally vasodilated diameter of arteriole (C, D) and venule (G, H) segments from day 7 to day 14. [*, ** indicate p<0.05 significant differences versus WT→WT and CX3R1^−/−^→WT, respectively, n = 4–7 mice per group].

WT mice reconstituted with CX3CR1^−/−^, CCR2^−/−^, or WT bone marrow (CCR2^−/−^→WT, CX3CR1^−/−^→WT, and WT→WT, denoted as marrow donor→host) were used to examine how loss of CX3CR1 and CCR2 specifically from BMCs affects inflammation-associated microvascular remodeling. Sample intravital microscopy images of microvascular remodeling in the DSFWC for the WT→WT, CCR2^−/−^→WT, and CX3CR1^−/−^→WT groups are shown in Figure 2A. No significant differences were seen in the number of arteriole or venule segments per window across experimental groups (8.7±1.0, 8.0±1.8, 6.3±2.9 arterioles, and 11.5±0.8, 11.3±1.0, 11.0±1.1 venules in WT→WT (n = 7), CX3CR1^−/−^→WT (n = 4), and CCR2^−/−^→WT (n = 6) mice, respectively) While there were differences in baseline arteriole and venule network structure, the mean starting arteriole diameter at day 7 did not vary across experimental groups (p>0.77, Figure 2B). As previously reported [8], [15], there was a significant increase (39.1±3.2 to 63.3±12.5 µm, p<0.001) in the mean diameter of individual arterioles from day 7 to day 14 in WT→WT control mice, but no significant change in diameter in either CX3CR1^−/−^→WT or CCR2^−/−^→WT mice (Figure 2B and 2C). Qualitative observations of the DSFWC suggested differential remodeling between smaller and larger arterioles, as previous reported [15]. Therefore, we split the analysis of arteriole segments to those above and below the aggregate median diameter at Day 7, 37.2 µm (Figure 3), similar to that previously performed by Doyle et al [15]. Scatter plots of Day 14 arteriolar diameter as a function of Day 7 arteriolar diameter are shown with a line of unity to illustrate the growth and regression of individual vessels (Figures 3A and 3B). For the larger arteriole segments, the percent change in maximal diameter was greatest in WT→WT controls. There was no significant remodeling in either CX3CR1^−/−^→WT or CCR2^−/−^→WT mice (Figure 3D). While there was a non-significant decrease in remodeling in CX3CR1^−/−^→WT mice for smaller arterioles compared to WT→WT controls, only CCR2^−/−^→WT mice reached significance (Figure 3C).

Venular lumenal remodeling was additionally considered. There were no significant differences in starting venular diameter at day 7, but a significant increase in diameter (p = 0.005) was observed in WT→WT mice from days 7 to 14 (Figure 2D). Venular remodeling in the CCR2^−/−^→WT mice from day 7 to 14 was not significant in terms of absolute diameter (80.0±11.5 to 93.6±19.0 µm, p = 0.09, Figure 2D). However, when examining the longitudinal percent change of venular diameter from day 7 to 14 (Figure 2E), which is the more useful metric for growth in the window chamber model, venular remodeling was significant. CX3CR1^−/−^→WT mice, however, exhibited significantly diminished venular remodeling compared to WT→WT and CCR2^−/−^→WT groups (Figure 2E). After splitting venules into those above and below median Day 7 diameter (81 µm), scatter plots showing growth and regression were generated (Figures 2E and 2F). The greatest impact of BMC-specific chemokine receptor knockout on venular remodeling occurred in the smaller segments, where CCR2^−/−^→WT mice had significantly greater percent increases in diameter than WT→WT control and CX3CR1^−/−^→WT mice (Figure 3G). Small venules in CX3CR1^−/−^→WT mice exhibited essentially no remodeling. No significant differences in large venule remodeling were observed between groups (Figure 3H).

## Discussion

The major finding from this study is that the influence of BMCs on microvascular remodeling is surprisingly complex and dependent upon multiple factors, including chemokine receptor expression (i.e. CCR2 and CX3CR1), the type of microvessel being considered (i.e. arteriole vs. venule), and initial microvessel diameter. We arrived at this conclusion by first demonstrating that the dorsal skinfold window chamber model is characterized by the infiltration of both CCR2+ and CX3CR1+ leukocytes (CD45+ cells) from Day 0 to 7 post-implantation (Figure 1D), followed by the lumenal growth of both the arterioles and venules from Day 7 to Day 14 (WT→WT group in Figures 2 and 3). With regard to arterioles, the expression of both CCR2 and CX3CR1 by BMCs was found to be required for complete lumenal growth (Figure 3C, 3D; Figure 2C). The abrogated growth of the arterioles in response to BMC-specific CCR2 deletion confirms an earlier study by our group [8]. When considering the small venules, BMC-specific CX3CR1 deletion also inhibited growth. However, in contrast, BMC-specific CCR2 deletion unexpectedly enhanced small venule growth (Figure 3G). Interestingly, while the recruitment of both CCR2+ and CX3CR1+ BMCs was complete by Day 7, mean Day 7 arteriolar and venular diameters were essentially the same for the CX3CR1^−/−^→WT, CCR2^−/−^→WT, and WT→WT groups. Thus, the influence of BMC-specific CCR2 or CX3CR1 deletion does not become evident until after BMC recruitment has stabilized. Together, the data suggest that targeting these characteristic chemokine receptors may provide an avenue for differentially modulating the extent of arterial versus venous remodeling therapeutically.

Additionally, it is important to understand the roles that CCR2 and CX3CR1 play in differentially recruiting resident and inflammatory monocyte populations when interpreting our results. In that CCR2-mediated recruitment is critical to inflammatory responses through migration and mobilization [4], [5] but not to resident monocytes [16], and CX3CR1-mediated recruitment is important to resident but less so to inflammatory monocytes [16], it suggests that BMC-specific knockout of CX3CR1 preferentially impairs resident monocyte recruitment. With our results denoting loss of CX3CR1 as additionally impairing venous remodeling, it suggests that resident monocytes additionally, if not preferentially, affect venous remodeling. One interesting hypothesis this raises is that this may be linked to this subset's venous patrolling [7] behavior and that the spatial differences in recruitment route or homing are related to spatial differences in physiological effect. While spatial differences in individual cell recruitment, could not be tracked in the current study due to the overall high density of BMC recruitment seen in the dorsal skinfold window chamber [8], recent work by Uchida et al [17] suggests that mononuclear cells involved in arteriogenesis and venogenesis during myocardial scar formation are routed from the capillaries and post-capillary venules to the interstitial space and to target vessels. This fits within the existing paradigm of leukocyte rolling and extravasation through post-capillary venules upon inflammatory stimulus in the microcirculation [18]. Therefore, CCR2 appears to be critical to arteriolar remodeling, but not venular remodeling, in the current data via preferential targeting toward arterioles, while CX3CR1 is essential to targeting to both venules and arterioles, whether through recruitment site or via post-extravasation migration.

The use of CX3CR1^−/−^→WT and CCR2^−/−^→WT mice enabled us to focus on the role of BMC-specific chemokine receptor function in inflammation associated microvascular remodeling arising from the implantation of a dorsal skinfold window chamber. This model provides several key advantages, but it also has limitations. First, one limitation of this approach is that experiments designed to “rescue” BMC-specific CX3CR1 and CCR2 signaling, such as through adoptive transfer of wild-type BM into the systemic circulation, transplantation of WT BMCs into the window chamber, or reconstitution of wild-type BM into CX3CR1^−/^ and CCR2^−/−^ mice, would be unlikely to yield credible results. This arises from the fact that adoptive transfer of small amounts of monocytes can yield systemic mobilization of endogenous monocyte subpopulations [19], [20], which would disrupt the temporally and spatially coordinated sequence of monocyte subpopulations necessary for vascular remodeling [14]. Similarly, inverse BMC reconstitution experiments, in which WT BMCS are transplanted into lethally irradiated CCR2^−/−^ and CX3CR1^−/−^ mice, would be incomparable due to the importance of autocrine signaling through endothelial cell expressed CX3CR1 [21], [22] and non-BMC sources of CCR2 signaling, as previously seen in the dorsal skinfold window chamber [8]. Second, the dorsal skinfold window chamber model used in this study is a variant from the standard window chamber model [23] in that only the epidermis was removed. This particular model results in a wound-healing like stimulus as evidenced by the extensive macrophage infiltration, granulation tissue build-up [8], and closure of the skin over the window after >14 days post implantation (data not shown). As such, during the initial surgical intervention, the pre-existing microvascular network is substantially altered, resulting in an expected rebalancing of blood flow and shear stress, a critical mediator of microvascular remodeling [14], [24], throughout the network both immediately post-implantation and during the wound healing process [25]. While the model has these limitations in terms of the complex interplay of stimuli driving the observed microvascular remodeling, the dorsal skinfold window chamber provides a unique advantage in allowing for longitudinal observation. Specifically, this allows for the assessment of how each individual vessel in the network remodels over time, thus negating the extensive heterogeneity of network structure between mice. Therefore, the current experiments and data provide a powerful means of testing the focused hypothesis of how loss of CCR2 and CX3CR1 from BMCs differentially affects remodeling in the microcirculation during an inflammatory, wound healing-like stimulus.

## Materials and Methods

### Ethics Statement

All animal studies were approved by the Institutional Animal Research Committee at the University of Virginia (Protocol #2785) and conformed to the American Heart Association Guidelines for the Use of Animals in Research.

### Bone Marrow Transplants

CCR2^−/−^ (B6.129S4-Ccr2^tm1lfc/J^), CX3CR1^−/−^ (B6.129P(Cg)-*Ptprc^a^ Cx3cr1^tm1Litt^* ^/LittJ^), and WT C57Bl/6 mice were procured from Jackson Laboratory (Bar Harbor, ME) for use as bone marrow donors. Each donor mouse provided enough bone marrow for 12 C57Bl/6 WT recipient mice. The recipient mice were sub-lethally irradiated via 2 doses of irradiation (550rad) to abrogate the host bone marrow. The mice were then reconstituted with donor marrow via tail vein injection and allowed 6 to 8 weeks for the donor marrow to engraft creating three groups (CCR2^−/−^→WT, CX3CR1^−/−^→WT, and WT→WT, denoted as marrow donor→host). This protocol has previously been shown to yield 95% reconstitution of donor genetics in circulating bone marrow derived cells [26].

### Dorsal Skinfold Window Chamber (DSFWC) Implantation

DSFWC implantations were performed as previously described [8]. Briefly, animals were anesthetizes with a ketamine, xylazine, and atropine cocktail (60, 4, and 0.2 mg/kg body weight, respectively, delivered i.p.). A circular section of skin from one layer of skin was excised under sterile conditions exposing the dermal microcirculation. The superficial fascia and panniculus carnosus were left intact. The exposed microvascular window was held in place using a surgically attached titanium dorsal skinfold window chamber (APJ Trading Co, Inc., Ventura, CA). After window chamber implantation, the exposed window was filled with sterile Ringer's solution and covered with a sterile coverglass. Animals received subcutaneous buprenorphine (0.1–0.2 mg/kg s.c.) for post-operative analgesia.

### Intravital Image Acquisition and Analysis for Assessing Microvascular Remodeling

Dorsal skinfold window chamber surgeries were performed on CCR2^−/−^→WT, CX3CR1^−/−^→WT, and WT→WT mice as described above and underwent intravital imaging as previous described [8], [15]. Mice were allowed 7 days after surgery to recover before image acquisition (day 7). Mice were anesthetized using inhaled isoflurane, the coverglass was removed, and the window was topically superfused with 10^−4^ M adenosine in sterile Ringer's solution to remove vessel tone, providing a consistent reference state. Therefore, any changes in arteriolar diameter can be attributed to changes in vessel structure. Within the window chamber, a region of interest (ROI) approximately 16 mm^2^ was chosen for longitudinal intravital imaging. Each ROI contained at least one large arteriole-venule pair and several smaller vessels. Intravital microscopic imaging was performed on a Zeiss Axioskop (Carl Zeiss Microimaging, Thornwood NY). Temperature was maintained throughout the duration of imaging. Arteriolar and venular diameter measurements were taken (ImageJ, U. S. National Institutes of Health, Bethesda, MD) between branch points at days 7 and 14 and the percent changes in diameter in diameter were calculated for each ROI containing ≥5 arteriole and venule segments. All arteriole and venule diameters, or changes in diameter from day 7 to 14 in a given window, were averaged. Mean values were,then, compared across animals within the experimental groups. Animals were euthanized upon completion of day 14 imaging by overdose of pentobarbital.

### Immunofluorescence Analysis of Window Chamber Tissue

A separate group of WT mice underwent DSFWC implantation for examination by immunofluorescence. DSFWC tissue was harvested either immediately (day 0) or 1, 7, or 14 days after implantation. To harvest tissue, mice were first euthanized with an overdose of pentobarbital and then perfused with Tris-buffered saline. The DSFWC tissue was excised and then frozen in Optimal Cutting Temperature Compound (OTC, Sakura Finetek USA, Torrance, CA). Tissue sections (5 microns in thickness) were cut with aid of a cryomicrotome. Slides containing DSFWC tissue cross sections were first blocked with solution containing 2% normal donkey serumand 0.1% saponin. Slides were then incubated overnight at 4°C with primary antibodies for CCR2 (CKR-2B goat polyclonal, sc-6228, Santa Cruz Biotechnology, Santa Cruz CA) at 1∶600 dilution, CX3CR1 (rabbit polyclonal, ab8021, Abcam, Cambridge MA) at 1∶150 dilution, Mac3 (M3/84 rat monoclonal, 550292, BD Biosciences Pharmingen, San Diego CA) at 1∶50 dilution, and CD45 (30-F11 rat monoclonal, 550539, BD Biosciences Pharmingen, San Diego CA) at 1∶200 dilution or with isotype control rat, rabbit, or goat IgG at 1∶200 dilution. Slides were then incubated for 90 minutes at room temperature with secondary antibodies: donkey anti-goat Cy3 conjugate (ab6949-100, Abcam, Cambridge MA) at 1∶800 dilution, donkey anti-rabbit Alexa Fluor 647 conjugate (A-31573, Invitrogen,Grand Island NY) at 1∶200 dilution, and donkey anti-rat Alexa Fluor 488 conjugate (A-21208, Invitrogen, Grand Island NY) at 1∶400 dilution. Slides were imaged on a Nikon TE200 (Nikon Instruments, Melville NY) microscope at 20× magnification. Each fluorescent color was thresholded to pre-determined values and area of CD45 positivity and colocalization with CCR2 and CX3CR1 positivity was determined with and without normalization to total cross sectional tissue area (ImageJ, U. S. National Institutes of Health, Bethesda MD).

### Statistics

All results are reported as ± standard deviation. All data were first tested for normality. Statistical significance was assessed by one and two-way ANOVA, followed by paired comparisons using the Holm-Sidak method. Significance was assessed at P<0.05.

## Supporting Information

Figure S1 **Isotype control immunolabeling for CD45, CCR2, and CX3CR1 in dorsal skinfold window chambers.** Representative images from day 7 window chambers demonstrating minimal non-specific immunolabeling for isotype IgG controls for CD45 (rat IgG primary), CCR2 (goat IgG primary), and CX3CR1 (rabbit IgG primary) compared to tissue labeled with primary antibodies. Scale bar is 50 µm.
(TIF)

Figure S2 **Immunolabeling for MAC3, CCR2, and CX3CR1 in dorsal skinfold window chambers.** Representative images from day 7 and 14 window chambers demonstrate that CCR2 and CX3CR1 positive cells are primarily Mac3+ macrophages. Density of Mac3+ cells suggest macrophages are the primary CD45+ cells in the window chamber tissue at days 7 and 14. Scale bar is 50 µm.
(TIF)
