# Supplementary figures and images for: Arteriolar and Venular Remodeling Are Differentially Regulated by Bone Marrow-Derived Cell-Specific CX3CR1 and CCR2 Expression
Source: PLoS One. 2012 Sep 24;7(9):e46312. doi: 10.1371/journal.pone.0046312 (PMC3454326; doi:10.1371/journal.pone.0046312)

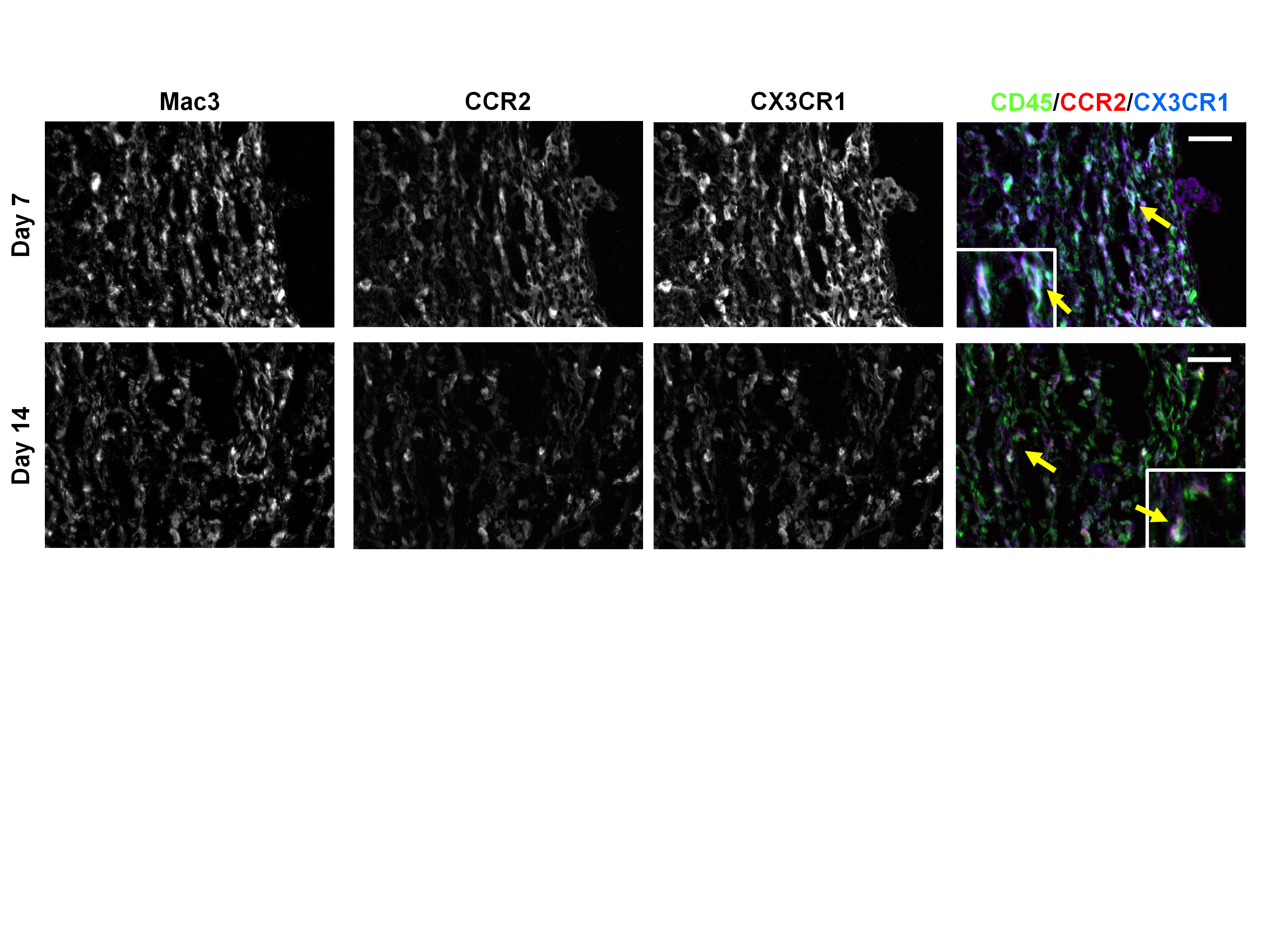

Supplement: Figure S2 — Immunolabeling for MAC3, CCR2, and CX3CR1 in dorsal skinfold window chambers. Representative images from day 7 and 14 window chambers demonstrate that CCR2 and CX3CR1 positive cells are primarily Mac3+ macrophages. Density of Mac3+ cells suggest macrophages are the primary CD45+ cells in the window chamber tissue at days 7 and 14. Scale bar is 50 µm. (TIF) [file pone.0046312.s002.tif]
